# Mathematical Modeling of Biofilm Structures Using COMSTAT Data

**DOI:** 10.1155/2017/7246286

**Published:** 2017-12-20

**Authors:** Davide Verotta, Janus Haagensen, Alfred M. Spormann, Katherine Yang

**Affiliations:** ^1^Department of Clinical Pharmacy, School of Pharmacy, University of California San Francisco, San Francisco, CA, USA; ^2^Novo Nordisk Foundation Center for Biosustainability, Technical University of Denmark, Kogle Alle 6, 2970 Hørsholm, Denmark; ^3^Department of Civil and Environmental Engineering, James H. Clark Center, Stanford University, Rm E250, 318 Campus Drive, Stanford, CA 94305, USA

## Abstract

Mathematical modeling holds great potential for quantitatively describing biofilm growth in presence or absence of chemical agents used to limit or promote biofilm growth. In this paper, we describe a general mathematical/statistical framework that allows for the characterization of complex data in terms of few parameters and the capability to (i) compare different experiments and exposures to different agents, (ii) test different hypotheses regarding biofilm growth and interaction with different agents, and (iii) simulate arbitrary administrations of agents. The mathematical framework is divided to submodels characterizing biofilm, including new models characterizing live biofilm growth and dead cell accumulation; the interaction with agents inhibiting or stimulating growth; the kinetics of the agents. The statistical framework can take into account measurement and interexperiment variation. We demonstrate the application of (some of) the models using confocal microscopy data obtained using the computer program COMSTAT.

## 1. Introduction

Biofilms are structured communities of bacteria enclosed in an extracellular matrix composed of polysaccharides, proteins, and extracellular DNA adherent to a surface [1]. Unlike planktonic bacteria, biofilms exhibit differences in metabolism, antibiotic tolerance, and ability to evade the immune system, making infections due to biofilms difficult to treat [2]. Biofilms are a main cause of acute and chronic infections, including foreign-body infections, otitis media, and urinary tract infections.

When a population of microorganisms organized in a biofilm grows, it is likely to pass over different phases. The growth may follow a period of dormancy, if the environmental conditions before the beginning of the growth are not optimal. Eventually, cells start to divide and structure, and the biofilm grows into a period in which the overall rate of cell division prevails over that of their death. Under favorable conditions, the growth may be considered to be unlimited (hence exponential) for some time, but eventually [3] physiological and physical limits such as (i) exhaustion of available nutrients, (ii) accumulation of inhibitory metabolites or end products, and (iii) exhaustion of space intervene. As a result, the growth rate decreases and the colony reaches its maximum size. Repeated or continual exposure to these environmental or physiological stressors can then result in a decline in the biofilm size [4].

In vitro biofilms are often used in studies concerning therapy, dealing with the reactions of bacterial populations to various agents: drugs, for example, antibiotics, or mutagens [5–8]. Those are applied externally to the biofilm structures and change their environment or directly eliminate (kill) the bacteria or decrease their reproductive capacity.

A variety of mathematical models have been used to describe bacterial growth in investigation of dynamics in environment depending only on the activity of the bacteria [9, 10]. Similarly, a number of models have been proposed to describe the action of different agents, in particular drugs and their interaction [11–13]. The objective of this paper is to obtain an integrated framework that provides models describing the different stages of biofilm growth, the action of different agents, and the simultaneous modeling of resulting kinetics for live and dead biofilm. The statistical issues associated with the use of these data, in particular the treatment of different sources of variability, are also addressed.

We demonstrate the use of the resulting general models using data obtained by means of confocal microscopy and COMSTAT [14]. COMSTAT takes the image stacks created by the confocal microscope as source data and produces up to ten image analysis features for quantification of biofilm structures which are output as one or more text files. The models we describe in this paper apply to univariate measurements: total biomass, area in a specific layer, average thickness, and volumes of microcolonies identified at the substratum (COMSTAT also obtains multivariate data, such as thickness distribution, which can be used to quantify the three-dimensional structures in the biofilm. The modeling of such data is the subject of current modeling investigation and will be reported in future communications.).

## 2. Methods

### 2.1. Mathematical Modeling

Modeling of biofilm growth requires the specification of three components. The first, a function *g*(·), describes the growth of the biofilm in absence of agents limiting or promoting growth. The second, a nonnegative function *h*(·), describes the interaction with the agents. The third, *C*(·), describes the temporal variation (kinetics) of the agents acting on the biofilm. The general model we consider expresses the rate of change of the biofilm as follows:

(1) dBtdt=gt,Bt±hCt,BtB0=B0,

where *t* is time, *B*(*t*) is biofilm amount present in the system at time *t*, *B*_0_ is the initial conditions or biofilm amount at *t* = 0, and *C*(*t*) is the concentration of agent at time *t*. The ± sign indicates that the model can describe either inhibition or stimulation.

#### 2.1.1. Biofilm Growth

A large number of models have been proposed to describe bacterial growth, for example, [15–17]. For the purpose of this paper, we only describe the simplest model for unlimited growth (exponential) and three semiempirical models for limited growth. Exponential biofilm growth assumes that the rate of growth is proportional to the amount of cells present in the system, and that there is no limitation to growth; thus

(2) $$\begin{array}{c} \frac{dB\left(t\right)}{dt}=g\left(\;t,B\left(\;t\;\right)\;\right)=k_{b}B\left(\;t\;\right), \end{array}$$

where *k*_*b*_ is the growth rate of the biofilm. The analytic solution of (2) is an exponential growth:

(3) $$\begin{array}{c} B\left(\;t\;\right)=B_{0}e^{k_{b}t} \end{array}$$

with doubling time, *t*_*d*_, being equal to ln⁡(2)/*k*_*b*_.

In general, exponential growth can only describe the early stages of biofilm growth. A number of semiempirical models can be used to take into account the decrease in proliferation that results from limitations of nutrient supply or mechanical constraints or metabolites accumulation. We consider the Logistic, Gompertz, and Bertalanffy models. The Logistic [18] takes the following form:

(4) $$\begin{array}{c} g\left(\;t,B\left(\;t\;\right)\;\right)=k_{b}B\left(\;t\;\right)\left(\;1-\frac{B\left(t\right)}{B_{max}}\;\right), \end{array}$$

where *B*_max_ is the maximum biofilm level that can be reached. The corresponding analytic solution is as follows:

(5) $$\begin{array}{c} B\left(\;t\;\right)=\frac{B_{max}B_{0}}{B_{0}+\left(B_{max}-B_{0}\right)e^{-k_{b}t}}. \end{array}$$

The Gompertz model [19–21] takes the following form:

(6) $$\begin{array}{c} g\left(\;t,B\left(\;t\;\right)\;\right)=k_{b}B\left(\;t\;\right)\ln\left(\;\frac{B_{max}}{B\left(t\right)}\;\right) \end{array}$$

with analytic solution:

(7) $$\begin{array}{c} B\left(\;t\;\right)=B_{max}e^{\ln\left(B_{0}/B_{max}\right)e^{-k_{b}t}}. \end{array}$$

Finally the Bertalanffy model [22] assumes that growth occurs proportionally to biofilm surface area, while biofilm loss is proportional to biofilm:

(8) $$\begin{array}{c} g\left(\;t,B\left(\;t\;\right)\;\right)=k_{b}B{\left(\;t\;\right)}^{2/3}-k_{d}B\left(\;t\;\right), \end{array}$$

where *k*_*d*_ is the death rate for biofilm. The solution of (8) is also sigmoidal in shape and tends to an asymptote as time increases, where the birth and death term balance each other out. A general version of the Bertalanffy model takes the following form [22]:

(9) $$\begin{array}{c} g\left(\;t,B\left(\;t\;\right)\;\right)=k_{b}B{\left(\;t\;\right)}^{\lambda }-k_{d}B{\left(\;t\;\right)}^{\delta }, \end{array}$$

where 0 < *λ* < *δ*. The Logistic equation is a special case of this model with *λ* = 1, *δ* = 2.

#### 2.1.2. Agents Interaction with Biofilm

The simplest model describing the interaction of bacterial biofilm with an agent assumes that the action of the agent is proportional to the product agent and biofilm:

(10) $$\begin{array}{c} h\left(\;C\left(\;t\;\right),B\left(\;t\;\right)\;\right)=\theta_{1}C\left(\;t\;\right)B\left(\;t\;\right), \end{array}$$

where *θ*_1_ now quantifies the interaction. For an inhibitory agent combining (10) with exponential growth (2) obtains

(11) $$\begin{array}{c} \frac{dB\left(t\right)}{dt}=k_{b}B\left(\;t\;\right)-\theta_{1}C\left(\;t\;\right)B\left(\;t\;\right). \end{array}$$

According to this model if the agent concentration is kept at constant level *k*_*b*_/*θ*_1_, the growth rate is zero. That concentration is the minimal concentration of agent killing bacteria or BIC. The BIC is frequently used to characterize bacterial growth in planktonic, in vitro experiments, and clinical settings (e.g., [23]). Note that if the growth rate is not exponential, the BIC is not a constant but depends on the current amount of biofilm. For example, for the Logistic model, incorporating an inhibitory agent action takes the following form:

(12) $$\begin{array}{c} \frac{dB\left(t\right)}{dt}=k_{b}B\left(\;t\;\right)\left(\;1-\frac{B\left(t\right)}{B_{max}}\;\right)-\theta_{1}C\left(\;t\;\right)B\left(\;t\;\right) \end{array}$$

and the BIC is given by the following equation:

(13) $$\begin{array}{c} BIC\left(\;B\left(\;t\;\right)\;\right)=\frac{k_{b}}{\theta_{1}}\left(\;1-\frac{B\left(t\right)}{B_{max}}\;\right) \end{array}$$

that shows how the BIC decreases as a function of *B*(*t*). Similarly, for the Gompertz model,

(14) $$\begin{array}{c} BIC\left(\;B\left(\;t\;\right)\;\right)=\frac{k_{b}}{\theta_{1}}\ln\left(\;\frac{B_{max}}{B\left(t\right)}\;\right). \end{array}$$

More complex models for the interaction agent/biofilm can be used, for example, threshold models, according to which the agent is effective if its concentration reaches a threshold,

(15) $$\begin{array}{c} h\left(\;C\left(\;t\;\right),B\left(\;t\;\right)\;\right)=\left\{\begin{array}{cc} \theta_{1}C\left(t\right)B\left(t\right) & \text{if }C\left(t\right)\geq \theta_{2}\\ 0 & otherwise, \end{array}\right. \end{array}$$

where the parameter *θ*_2_ is the threshold; or models following saturation kinetics similar to what is used in, for example, pharmacodynamics [13],

(16) $$\begin{array}{c} h\left(\;C\left(\;t\;\right),B\left(\;t\;\right)\;\right)=\frac{\theta_{1}C\left(t\right)}{\theta_{2}+C\left(t\right)}B\left(\;t\;\right), \end{array}$$

where now, for large concentrations of agent, the killing rate asymptotes to *θ*_1_*B*(*t*). In presence of two agents, *C*_1_(*t*) and *C*_2_(*t*), models (10) and (16) increase in complexity, as the agents can affect the killing rate in different ways. For example, the linear model (10) can be generalized as follows:

(17) $$\begin{array}{c} h\left(\;C_{1}\left(\;t\;\right),C_{2}\left(\;t\;\right),B\left(\;t\;\right)\;\right)=\left(\;\theta_{1}C_{1}\left(\;t\;\right)+\theta_{2}C_{2}\left(\;t\;\right)\;\right)B\left(\;t\;\right) \end{array}$$

or

(18) hC1t,C2t,Bt=θ1C1t+θ2C2t+θ3C1tC2tBt,

where *θ*_3_ is a killing rate for the agents' interaction. When saturation kinetics are present, as is the case for the single agent model (16), a number of possibilities arise, generating additive, synergistic, and antagonistic models often applied in pharmacodynamic, enzymology, or binding experiments [12]. For example, incorporating a model for competitive interaction between two agents yields

(19) hC1t,C2t,Bt=C1t/C1,50+αC2t/C2,501+C1t/C1,50+C2t/C2,50Bt,

where if *α* > 0 the model shows additivity, and when *α* = 0 it reduces to the competitive antagonism model and yields

(20) hC1t,C2t,Bt=C1tC1t+C1,501+C2t/C2,50Bt.

#### 2.1.3. Agent Kinetics

A mathematical representation of the agents' kinetics is needed to incorporate their action on the biofilm. This in general does not present particular difficulties. Analytical solutions or sets of differential equations can be used to do so [24, 25]. As an alternative one can use “model independent” representations of the data, such as smoothing splines [26], or, when the measurement error in the kinetics data is low an even simpler linear interpolant as we do in the examples reported below.

#### 2.1.4. Modeling Dead Biofilm

Confocal microscopy data allow the measurement of both live and dead cells (see Data section). Indicating by *D*(*t*) the dead biofilm present in the system at time *t*, its rate of generation can be directly obtained from the growth equations reported above by identifying the loss or gain in live cells due to the presence of the agent. For the Logistic growth rate equation (4), this yields

(21) $$\begin{array}{c} \frac{dD\left(t\right)}{dt}=k_{b}\frac{B{\left(t\right)}^{2}}{B_{max}}\pm h\left(\;C\left(\;t\;\right),B\left(\;t\;\right)\;\right), \end{array}$$

and, for the generalized Bertalanffy model equation (8), an inhibitory agent obtains

(22) $$\begin{array}{c} \frac{dD\left(t\right)}{dt}=k_{d}B{\left(\;t\;\right)}^{\delta }+h\left(\;C\left(\;t\;\right),B\left(\;t\;\right)\;\right). \end{array}$$

For the Gompertz model, the growth equation does not lead directly to express a rate of cell loss since it only expresses a decrease of the growth rate *k*_*b*_ inversely proportional to biofilm growth. A possibility is to express dead biofilm as follows:

(23) $$\begin{array}{c} D\left(\;t\;\right)=B_{0}e^{k_{b}t}-B\left(\;t\;\right), \end{array}$$

the difference between what would result from exponential growth and the actual biofilm level.

#### 2.1.5. Modeling Post-Plateau Biofilm Decrease

To account for effects leading to a post-plateau decline in the biofilm size [4], one can introduce a hypothetical endogenous variable, *X*, that is reduced proportionally to the amount of biofilm present [27, 28].

(24) $$\begin{array}{c} \frac{dX\left(t\right)}{dt}=-k_{l}B\left(\;t\;\right),\;X\left(0\right)=1, \end{array}$$

where *k*_*l*_ is the rate of reduction. *X*(*t*) is arbitrarily set to 1 at time zero; it is positive as *B*(*t*) increases, but eventually it becomes negative (as it can be seen by the relationship *X*(*t*) = 1–*k*_*l*_∫_0_^*t*^*B*(*t*)*dt*). The growth rate of biofilm takes the following form:

(25) $$\begin{array}{c} g\left(\;t,B\left(\;t\;\right),X\left(\;t\;\right)\;\right)=g\left(\;B\left(\;t\;\right)\;\right)X\left(\;t\;\right), \end{array}$$

where *g*(*B*(*t*)) is given by (4) or (6). For example, for Logistic growth,

(26) $$\begin{array}{c} \frac{dB\left(t\right)}{dt}=\left[\;k_{b}B\left(\;t\;\right)\left(\;1-\frac{B\left(t\right)}{B_{max}}\;\right)\;\right]X\left(\;t\;\right). \end{array}$$

The right side of equations (25) and (26) becomes negative as time progresses and leads *B*(*t*) to asymptote to zero (*X*(*t*) asymptotes to a negative value). The main limitation of the model is that for any value of *k*_*l*_ the biofilm always asymptotes to 0. To avoid this the model can be modified as follows:

(27) $$\begin{array}{c} \frac{dX\left(t\right)}{dt}=k_{p}-k_{q}X\left(\;t\;\right)-k_{l}B\left(\;t\;\right),\;X\left(0\right)=\frac{k_{p}}{k_{q}}, \end{array}$$

where *k*_*p*_ and *k*_*q*_ are production and elimination rates of the endogenous substance. Equations for dead cells are obtained as before.

We remark that especially in in vivo systems one can observe pre- or post-plateau appearance of cyclical growth, associated with seeding and dispersal [29, 30]. To model these situations one could use a time-varying *B*_max_ that changes value after a pure time-delay or depending on a threshold biofilm value.

#### 2.1.6. Modeling Dormancy

Biofilm growth can follow an initial period of dormancy that can be expressed easily using a (pure) lag time. The growth rate becomes

(28) $$\begin{array}{c} g\left(\;t,X\left(\;t\;\right)\;\right)=\left\{\begin{array}{cc} 0 & t<t_{lag}\\ g\left(B\left(t-t_{lag}\right)\right) & otherwise, \end{array}\right. \end{array}$$

where *t*_lag_ is the lag time.

### 2.2. Statistical Modeling and Model Selection

Biofilm data usually consist of several measurements made on a number of growing “cells” on different occasions. The measurements present different levels of random variation: among measurements within a given cell (intracell variation), among cells (intercell variation), and interoccasion. A hierarchical nonlinear mixed effect model [31] can be used to represent such data. According to this model the *i*th observation from the *j*th cell and *k*th experiment, *y*_*ijk*_, takes the following form:

(29) $$\begin{array}{c} y_{ijk}=V\left(\;t_{ijk},h\left(\;\delta ,\eta_{j},\nu_{k}\;\right)\;\right)+\varepsilon_{ijk}, \end{array}$$

where *t*_*ijk*_ is the corresponding sampling time, *V* is a nonlinear function describing the relationship between time, parameters, and predicted response, and *h*(*δ*, *η*_*j*_, *ν*_*k*_) describes the parameters values for each cell, which depend on *δ*, a fixed effect mean parameter vector, a vector of intercell random effect parameters, *η*_*j*_, and a vector of intercell random effect parameters, *ν*_*k*_, with mean zero and a certain, typically multivariate normal, distribution with variance-covariance matrix *Ω*. The random effect *ε*_*ijk*_ indicates intracell variability (e.g., measurement error) typically assumed (multivariate) normally distributed with mean zero and covariance matrix Σ, which could depend on *δ*, *η*_*j*_ (we omit details for simplicity) and an unknown parameter vector *σ*. Popular methods used to fit mixed effect models to data are implemented in the computer programs such as NONMEM [32] and NLME [33], which allow the computation of corresponding second-order statistics for the estimates, in particular the large sample variance-covariance matrix of the parameters *δ*, individual parameters as empirical-Bayes estimates, and corresponding predictions, conditional on the estimates for *δ*, *Ω*, Σ.

To select between different models one can use statistical model selection criteria together with the usual graphical displays based on predictions, observations, and residuals. Some statistical model selection criteria are the Hannan-Quinn (HQ) [34] and Akaike (AKA) [35]; these criteria penalize the likelihood of the model proportionally to the number of parameters in the model, thus penalizing the selection of models with ‘too many' parameters. The HQ is the most conservative; it uses twice the log of the number of observations times the number of parameters as penalty, while the AKA uses twice the number of parameters.

### 2.3. Data

*Pseudomonas aeruginosa* PAO1 tagged with green fluorescent protein (GFP) were studied in flow-chamber experiments described in [36]. In brief, biofilms were grown at 30°C in flow chambers. Each flow chamber was inoculated with 250 *μ*L of an overnight culture of PAO1 diluted to an OD_600_ of 0.05 and left without flow for one hour. After one hour, flow was started with minimal media at a flow rate of 20 ml/h using a peristaltic pump (Watson Marlow 205 S). After cultivation for 24 or 72 hours, flow was stopped and minimal media were replaced with an antibiotic flask containing the desired concentration of either Meropenem (MEM) or Tobramycin (TOB) where administered as an intermittent bolus. This antibiotic flask is connected to bubble traps and the flow chambers containing the cultivated PAO1 biofilms. Flow was restarted and minimal media were pumped from the dilution flask to the antibiotic flask to the flow chambers at a constant rate calculated to mimic the elimination rate constant of the antibiotic. MEM and TOB were obtained from the pharmacy of the University of California San Francisco Medical Center [37]. Concentration-time profiles were based on previously described PK parameters of MEM and TOB from healthy volunteers and patients with cystic fibrosis [38, 39]. The target MEM peak concentration based on human population values was computed to be 107.53 mg/L with a *t*_1/2_ = 0.893 h. The target TOB peak concentration, based on a dose of 10 mg/kg in a 70 kg adult, was 32.79 mg/L with an associated *t*_1/2_ = 2.75 h. Samples were analyzed by liquid chromatography-tandem mass spectrometry (LC-MS/MS) [40].

Microscopic observations of the flow cells were completed using a Leica TCS SP2 confocal laser scanning microscopy (CLSM) with an argon/krypton laser and detectors and filter sets for simultaneous monitoring of GFP (excitation, 488 nm; emission, 517 nm) for live cell staining and propidium iodide (excitation, 543 nm; emission, 565 nm) for dead cell staining. Images were acquired at approximately 1 *μ*m intervals in the *z* direction down through the biofilm. Each channel of the flow chamber was randomly imaged at two separate locations per time point. CLSM images were analyzed using COMSTAT [14].

## 3. Results

**Figure 1:**
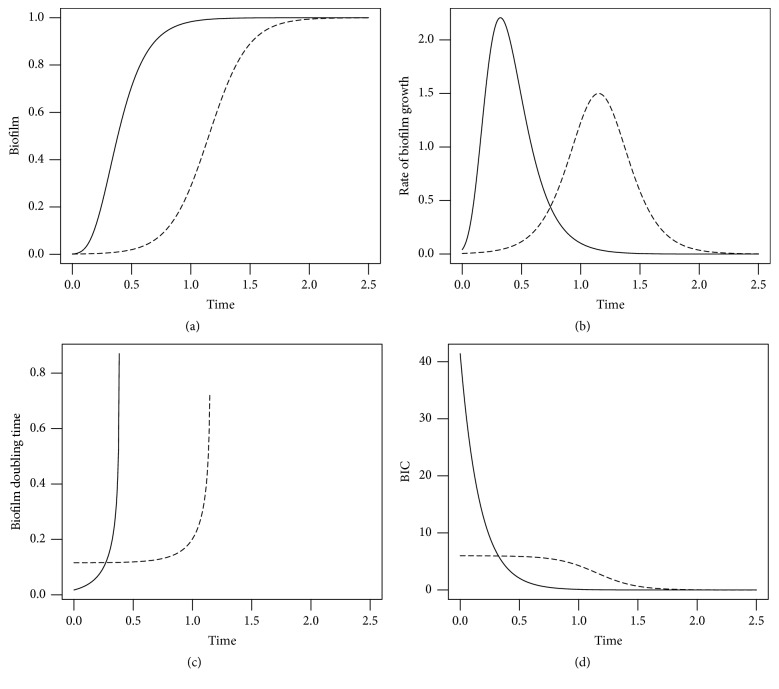
Biofilm growth models: simulation. (a) Biofilm growth according to the Gompertz (solid line) and Logistic (dashed line) models. (b) Corresponding time derivatives. (c) Corresponding biofilm doubling time. (d) Corresponding BIC. Parameter values: *B*_0_ = 0.001, *k*_*b*_ = 6, *B*_max_ = 1, *θ*_1_ = 1. (Biofilm and time are in arbitrary units.)

*Biofilm Growth Models: Simulation*.  Figure 1(a) shows a plot of the Gompertz and Logistic models (see (7) and (5)) with parameters values *B*_0_ = 0.001, *k*_*b*_ = 6, *B*_max_ = 1, and time in arbitrary units.  Figure 1(b) shows their corresponding time derivative (i.e., the biofilm overall rate of change), and Figure 1(c) the biofilm doubling time. Doubling time is not a constant, as for the exponential model equation (3), but is instead expressed by the following relationships:

(30) $$\begin{array}{c} t_{d}=\frac{\ln\left(\ln\left(2\right)/e^{-k_{b}t}+1\right)}{k_{b}}, \end{array}$$

for the Gompertz, and

(31) $$\begin{array}{c} t_{d}=-\frac{1}{k_{b}}\ln\left(\;\frac{1}{2}-\frac{B_{0}}{2\left(B_{max}-B_{0}\right)}e^{k_{b}t}\;\right) \end{array}$$

for the Logistic model. Note how the doubling time is only defined up to the time for which *B*(*t*) = *B*_max_/2, where it tends to infinity.  Figure 1(d) shows the BIC for the two models: note how the BIC is a decreasing function of *B*(*t*), because intrinsic growth rate decreases as *B*(*t*) reaches the asymptote *B*_max_.

**Figure 2:**
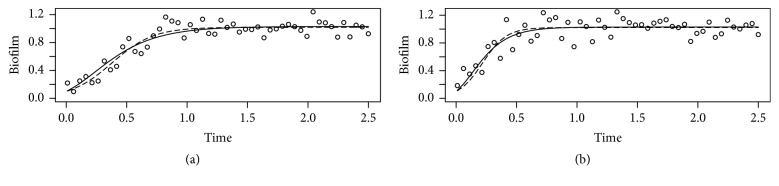
Simulated Data Example 1. Gompertz and Logistic models data fit. Circles, data simulated using a Logistic (a) or Gompertz (b) model with added random normally distributed noise; parameter values: *B*_0_ = 0.001, *k*_*b*_ = 6, *B*_max_ = 1. Solid lines and dashed lines: Gompertz and Logistic model fit to the data, respectively. See also legend to Figure 1.

*Simulated Data Example 1*. Different growth models are often difficult to discriminate when fitted to data.  Figure 2 shows data simulated using a Logistic (Figure 2(a)) or Gompertz (Figure 2(b)) model. The data are generated by adding random normally distributed noise with mean 0 and standard deviation 0.1 to the predictions; parameter values are *B*_0_ = 0.001, *k*_*b*_ = 6, *B*_max_ = 1. Visually, the fits are difficult to distinguish, and they are undistinguishable according to the Akaike criterion. There is difference in Akaike value between the two models of 0.8 for Figure 2(a) and 1.2 for Figure 2(a).

The Bertalanffy model equation (8) can describe rather different kinetics than the Logistic (and Gompertz) depending on the values of its parameters. In general one cannot reproduce data generated by generalized Bertalanffy process using either a Gompertz or Logistic equation.

**Figure 3:**
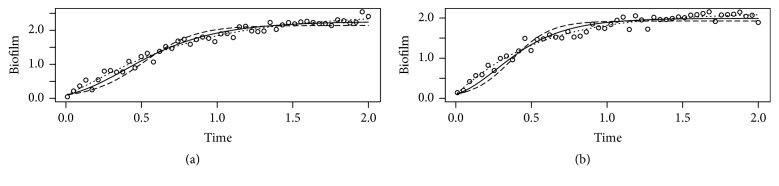
Simulated Data Example 2. Bertalanffy versus Gompertz and Logistic models data fit. Circles, data simulated using a Bertalanffy model with added random normally distributed noise. Parameter values: *k*_*b*_ = 4, *k*_*d*_ = 2, *λ* = 0.25, *δ* = 1; *k*_*b*_ = 4, *k*_*d*_ = 2, *λ* = 0.1, *δ* = 1 (b). Solid lines, dashed lines, and thin dashed lines: Bertalanffy, Gompertz, and Logistic model fit to the data, respectively. See also legend to Figure 1.

*Simulated Data Example 2*.  Figure 3 shows an example where data are simulated using the Bertalanffy model with *k*_*b*_ = 4, *k*_*d*_ = 2, *λ* = 0.25, *δ* = 1(Figure 3(a)) or *k*_*b*_ = 4, *k*_*d*_ = 2, *λ* = 0.1, *δ* = 1(Figure 3(b)), adding normally distributed error with mean 0.0 and standard deviation 0.1. Note the misfit of the Gompertz (dashed line) and especially Logistic model (thin dashed line) when compared to the Bertalanffy fit (solid line). The differences in the objective functions corresponding to the fits are in this case statistically significant according to the Akaike criterion.

**Figure 4:**
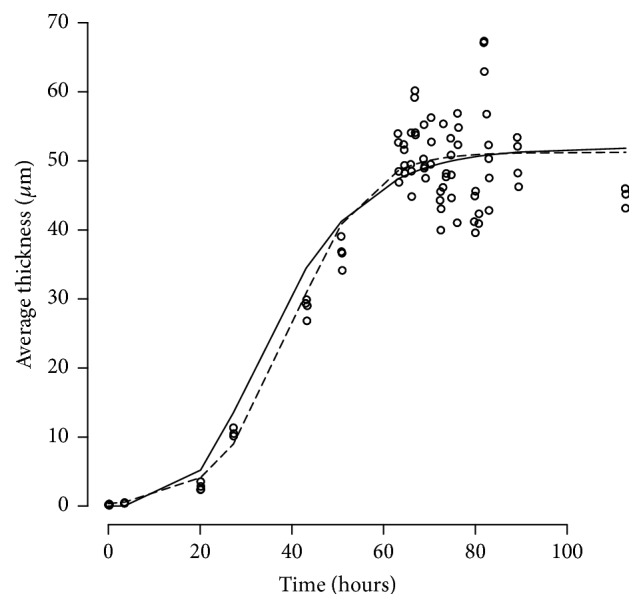
Real Data Example 1. Circles, average thickness data with superimposed predictions of the fits of a Logistic (dashed line) and Gompertz (solid line) models.

*Real Data Example 1*.  Figure 4 shows the result of the fits of the Logistic and Gompertz models to average thickness control data. Note how the average thickness increases and then reaches a plateau. The Logistic (dashed line) and Gompertz (solid line) fits estimate a similar value for *B*_max_ but have slightly different shapes. The objective function values for the Logistic and Gompertz models were 373.50 versus 375.35, indicating a statistically insignificant, slightly better fit for the Logistic model. According to the Logistic model the maximum biofilm growth, (*B*_max_), was 52.1 (*μ*m) with a birth rate (*k*_*b*_) of 0.051 (1/h). According to the Gompertz model the maximum biofilm growth, (*B*_max_), was 53.2 (*μ*m) with a birth rate (*k*_*b*_) of 0.049 (1/h).

**Figure 5:**
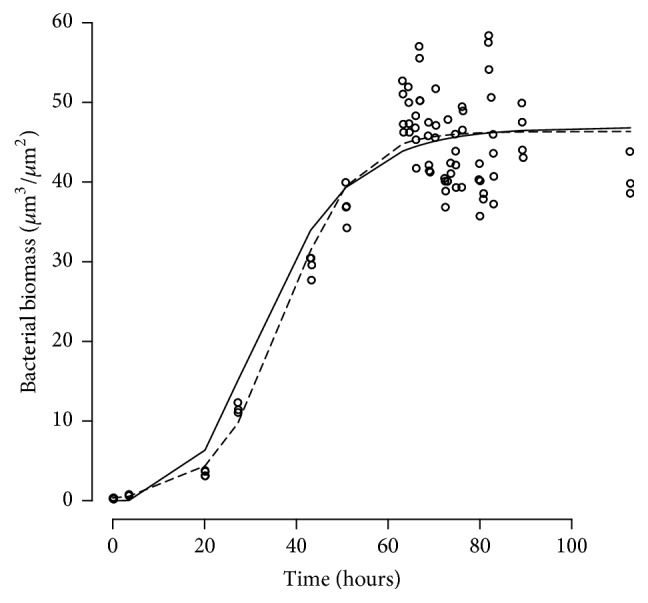
Real Data Example 2. Circles, biomass data with superimposed the predictions of the fits of a Logistic (dashed line) and Gompertz (solid line) models.

*Real Data Example 2*.  Figure 5 shows the result of the fits to biomass data collected in the same cells. The Logistic (dashed line) and Gompertz (solid line) fits estimate a similar value for *B*_max_, the maximum growth level, and have slightly different shapes before asymptote toward *B*_max_. The objective function values for the Logistic and Gompertz models were 585.86 versus 585.93, indicating that the models cannot be discriminated based on the fit. According to the Gompertz model, the maximum biofilm growth, (*B*_max_), was 42.5 *μ*m^3^/*μ*m^2^ with a birth rate (*k*_*b*_) of 0.051.3 1/h.

**Figure 6:**
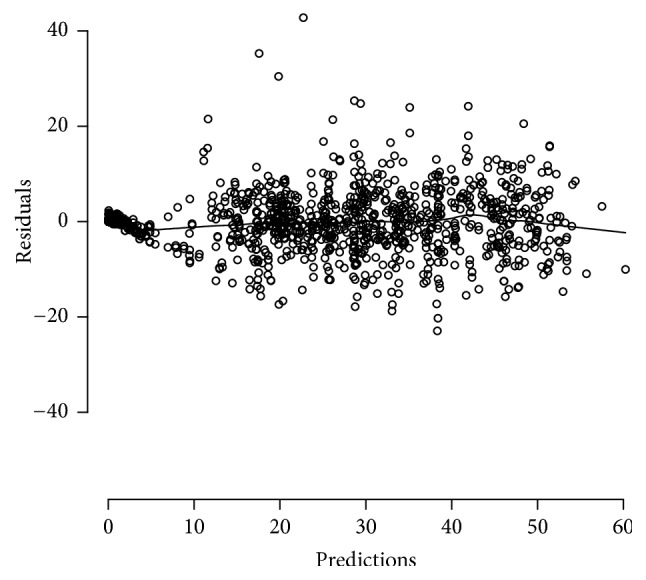
Real Data Example 3: model residuals plot. Gompertz model fit to multiple administration Meropenem (MEM) and Tobramycin (TOB) data (see also legend to Figure 8). Open circles, nonlinear mixed effect model weighted residuals versus predictions; solid line, R function supsmu fit to the weighted residuals.

*Real Data Example 3*. This demonstrates the use of a nonlinear mixed effect model on a complex data set that includes controls, single-dose administration of MEM at TOB at 24 hours (monoexponential decay), triple dose (monoexponential decay) of both drugs at 74 hours, and a combined single-dose infusion of MEM and single dose (monoexponential) or TOB at 72 hours.  Figure 6 shows the plot of the weighted residuals versus time for the fit to data; the solid lines show the R [41] function supsmu fit to the residuals. Note the overall lack of trends (a trend in the residuals would indicate a misfit of the model to the data), although there is a minor trend at the lowest and highest prediction values.

**Figure 7:**
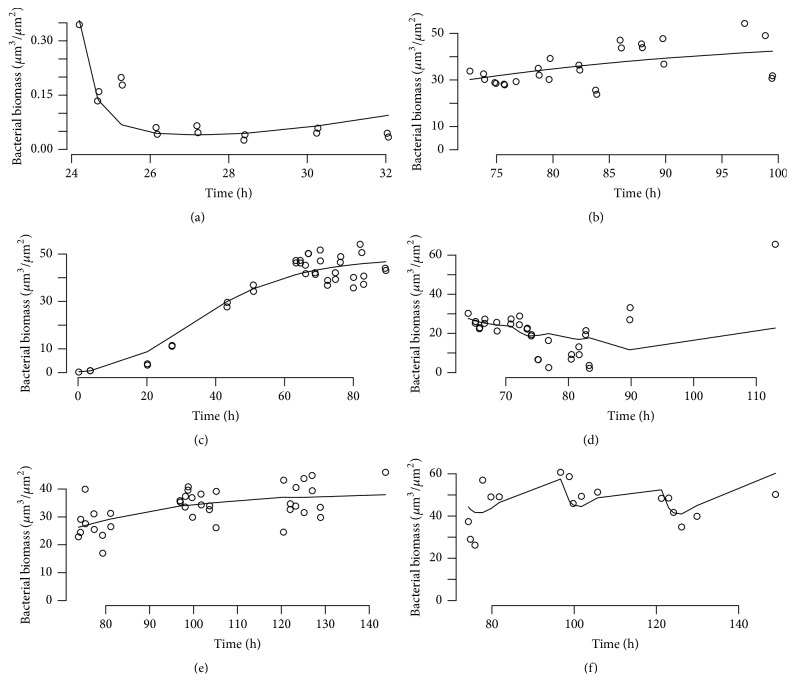
Real Data Example 3: observations and model predictions. Gompertz model fit to multiple administration MEM and TOB data (see text). In all panels: open circles, observations; solid lines, model predictions. Panels: top to bottom, right to left: 10% best fit, 25%, 40%, 60%, 75%, and 90% (worst fit).

**Figure 8:**
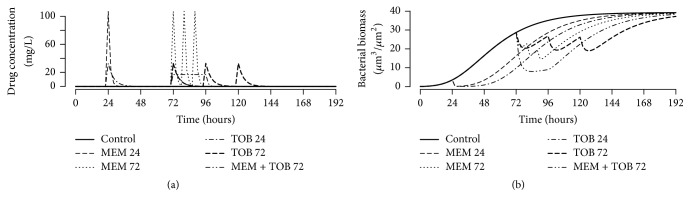
Real Data Example 3: simulation using the fitted model. (a) MEM and TOB administrations. (b) The corresponding predictions by the model using the estimates reported in Table 1.

Figure 7 shows plots of the observations (open circles) and empirical-Bayes predictions by the model (solid line) for a selection of the data sets. The data are selected based on the objective function value of the individual fit to each data set. Top to bottom, right to left they correspond to the 10% (best), 25%, 40%, 60%, 75%, and 90% (worst) fit of the model.  Table 1 reports the parameter estimates, showing the partition of the variability of the parameter estimates.  Figure 8 shows a simulation based on those parameter estimates: drug kinetics in Figure 8(a) and the corresponding biomass vsersus time plot in Figure 8(b).

**Figure 9:**
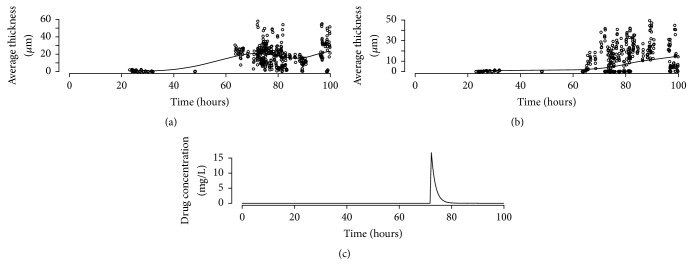
Real Data Example 4. (a) Live biofilm average thickness. (b) Dead biofilm average thickness. (c) Drug administration profile. All panels: circles, observations; solid line, model predictions.

*Real Data Example 4*. Finally, as an example of a combined fit to live and dead cells Figure 9(a) shows the fit of the Logistic growth model incorporating drug effect according to (15) to the live biofilm average thickness data pooled from twelve experiments; Figure 9(b) shows the fit of to the dead cells; Figure 9(c) shows the pharmacokinetic profile imposed during in the experiments. Note how the model follows quite well the decrease in average thickness following the drug administration and tracks the corresponding increase of dead cells despite the large variability for both live and dead data measurements. The estimate of the killing rate (*θ*_1_) in this example is 0.00243 (1/h/*μ*m).

## 4. Discussion

In this paper, we describe general mathematical models that can be used to quantify biofilm growth and interaction with different agents inhibiting or stimulating growth. The models are semiempirical in nature, in the sense that they do not include a detailed physiological description but allow the quantification of biofilm growth and the interaction with growth-inducing or growth-reducing agents.

With regard to biofilm growth, we show that the commonly used BIC (the minimal drug concentration that inhibits growth) is not a constant when growth is limited, which is almost always the case with biofilms, but depends on the actual amount of biofilm and corresponding growth status.

We show how different growth models are often difficult to distinguish when fitted to data. This is due to the inherent flexibility of the models and also related to the sampling frequency and noise level present in the data. In the examples reported here, the sampling is relatively frequent. But in general, other experimental conditions might allow more frequent sampling and therefore the identification of more complex growth models and/or more complex models for the response to agents affecting biofilm growth.

The main advantage of the use of a mathematical model is that it can provide a characterization of complex data in terms of few parameters. As an example, Table 1 shows how a model with just five parameters characterizes seven distinct experiments. In addition, a nonlinear mixed effect model allows the identification not only of the parameters characterizing the growth and growth inhibition but also of the sources of variability present in a particular experimental situation. This is important because it allows the comparison of the quality of the data arising from different experimental conditions, and it suggests where experimental variation might need to be reduced. The main advantages of using a nonlinear mixed effect model are its statistical elegance and its efficiency. The main disadvantages are the many distributional assumptions involved in the formulation of the model and the computational burden involved in estimating the likelihood of the observations. A simple alternative, which takes advantage of the relatively abundance of data in in vitro experiments, is to fit data from each cell and experiment independently, obtaining the corresponding parameter estimates, which are then used to characterize the distribution of the parameters in the collection of experiments. This requires no specialized software and might be the most appealing method of choice in many situations.

The most important function of a mathematical model is the characterization of the process underlying data generation (rather than random variation associated with data collection). The models we describe represent a summary of models already present in the literature but also include potentially useful new models describing the kinetics of dead cells, together with models describing agents and agent interaction with growth. Taken together, they can allow the representation of a vast array of biofilm data.

## Figures and Tables

**Table 1:** Biofilm growth parameters and kills rates associated with MEM and TOB.

Parameter	Parameter estimate	Interchannel variability (%)
*B*_max_ (*μ*m^3^/*μ*m^2^)	39.5 (1.34)	21.8
*k*_*b*_ (1/h)	0.0425 (0.0406)	11.1
*k*_1_ (1/h/cu)	0.00301 (0.00011)	14.8
*k*_2_ (1/h/cu)	0.00352 (0.000168)	20.9
*k*_12_ (1/h/cu^2^)	0.000473 (0.000083)	7.1

*Residual Variability σ* (*μ*m^3^/*μ*m^2^)	8.02	-

*B*_max_, maximum biofilm growth (in absence of drug); *k*_*b*_, biofilm growth rate (in absence of drug); *k*_1_, MEM kill rate; *k*_2_, TOB kill rate; *k*_12_, drug interaction kill rate (cu, concentration units, 1000*∗*ng/mL). Standard errors for the estimated parameters are in parenthesis.
